# 

**Published:** 1890-01

**Authors:** 


### CONTENTS.

### COMMUNICATIONS.

Diseases of the Antrum........ 5
New Italian Review on Hygiene........ 12
Tumors........ 13
Death from Chloroform........ 16
The Physical Changes Produced by Decay........ 17
Chapped Hands........ 21

### SELECTIONS.

Some Discussions on Tuberculosis, etc........ 22
The Scientific Uses of Very High Towers........ 25
Extract from Clinical Lecture, Primary Dentition........ 28
Practical Notes........ 31
Department of Hygiene at the University of Pennsylvania........ 34
The Native Egyptian as a Subject of Surgical Operation........ 35
Death after Ether Anæsthesia........ 35
Administration of Anæsthetics........ 36
Do Not Waste Alcohol........ 37
Letter from Berlin........ 38
The New Hypnotic-Sulphonal........ 41
The Use of Antiseptics in Dentistry........ 43
Source of Colors........ 44
Beer Compared with other Alcoholics........ 45
Nitrous Oxide Gas—The Phonograph as a Reporter........ 46
Statistics of Breathing—A New Cure for Toothache........ 47
A Bungling Chemist's Discovery—Test for Blood........ 48
Tenth International Medical Congress, Berlin, 1890........ 49

### CORRESPONDENCE.

Northern Indiana Dental Society........ 51

### EDITORIAL.

Removal........ 52
Odontological Society of Cincinnati........ 52
Students' Society........ 53
Dr. William M. Hunter........ 54
Obituary........ 55
National Association of Dental Faculties........ 56

